# FDG uptake reflects an immune‐enriched subtype of thyroid cancer: Clinical implications of imaging‐based molecular characterization

**DOI:** 10.1002/cam4.6350

**Published:** 2023-07-19

**Authors:** Hoon Young Suh, Hongyoon Choi, Sun Wook Cho, Jin Chul Paeng, Gi Jeong Cheon, Young Joo Park, Keon Wook Kang

**Affiliations:** ^1^ Department of Nuclear Medicine Seoul National University Hospital Seoul Republic of Korea; ^2^ Department of Molecular Medicine and Biopharmaceutical Sciences, Graduate School of Convergence Science and Technology Seoul National University Seoul Republic of Korea; ^3^ Institute of Radiation Medicine, Medical Research Center Seoul National University College of Medicine Seoul Republic of Korea; ^4^ Department of Internal Medicine Seoul National University Hospital Seoul Republic of Korea; ^5^ Department of Internal Medicine Seoul National University College of Medicine Seoul Republic of Korea; ^6^ Cancer Research Institute Seoul National University Seoul Republic of Korea; ^7^ Institute on Aging Seoul National University Seoul Republic of Korea; ^8^ Genomic Medicine Institute, Medical Research Center Seoul National University College of Medicine Seoul Republic of Korea; ^9^ Department of Biomedical Sciences Seoul National University College of Medicine Seoul Republic of Korea

**Keywords:** 2‐deoxy‐2‐fluoro‐D‐glucose, thyroid neoplasms, tumor microenvironment, glycolysis, glucose transport proteins

## Abstract

**Introduction:**

Iodine and FDG uptakes have been established as methods to define the biological properties of thyroid cancer. As various cells in the tumor microenvironment (TME) affect tumor metabolism, we investigated the association between glucose metabolism in thyroid cancer and the TME using transcriptomic analyses.

**Methods:**

We used F‐18 FDG PET and RNA sequencing data of thyroid cancer to find associations between TME cell types and glucose metabolism. In addition, publicly available single‐cell RNA sequencing data of papillary thyroid cancer was used to investigate glucose metabolism in cell types of the TME. The correlations between the FDG uptake and biological properties of the TME, including glucose metabolism and tumor differentiation score (TDS) were evaluated. Estimation of the proportions of immune and cancer cells (EPIC) was performed. The biological properties of each cell type were also assessed in the single‐cell RNA sequencing data.

**Results:**

FDG uptake showed a positive correlation with the enrichment score of macrophages and glycolysis activity. In single‐cell RNA sequencing, immune cells had both high glucose transporters (GLUTs) and glycolysis signatures, while thyrocytes including cancer cells showed relatively low GLUTs and glycolysis signatures, suggesting that FDG uptake mainly occurred in immune cells of the TME. Moreover, the high GLUTs of myeloid cells were negatively associated with TDS.

**Conclusions:**

Our findings suggest that thyroid cancer with high FDG uptake can be mediated by enriched immune cells of the TME. We suggest that FDG uptake in thyroid cancer could be a marker for the immune‐rich type and provide clinical implications for treatment stratification.

## Graphical abstract

Thyroid cancers can be divided into radioactive iodine (RAI) refractory and RAI effective cancers depending on the degree of iodine uptake. In addition, thyroid cancers are further classified as immune cold and immune hot because FDG uptake represents the immune abundancy of the thyroid cancer microenvironment.

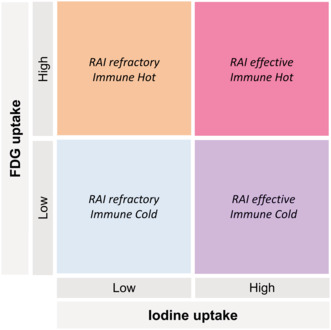

## 1 INTRODUCTION

Radioactive iodine (RAI) and 2‐deoxy‐2‐[^18^F]fluoro‐d‐glucose (FDG) have been widely used for the evaluation of thyroid cancer. Thyroid cancer with poor differentiation has been shown to have low iodine avidity and high FDG avidity.^1^, ^2^ This ‘flip‐flop phenomenon’ was explained as the dedifferentiation of cancer cells resulting in the capacity to accumulate iodine decreasing while the demand for glucose increases.^3^ While previous studies have focused on thyroid cancer cells themselves, the understanding of tumor metabolism should not be limited to cancer cells but should extend to the tumor microenvironment (TME), which plays a critical role in the cell‐selective partitioning of nutrients.^4^, ^5^

The TME plays an important role in regulating cancer progression and response to treatment. The presence of immune cells, including lymphocytes and macrophages, within or near cancer supports the immune‐related microenvironment of thyroid cancer.^6^ Generally, immune cell‐enriched tumors are prone to demonstrate good immunotherapy responsiveness compared to immune cold types, although the effects of various cell types in the TME are complex.^7^ In thyroid cancer, tumor‐infiltrating immune cells have dual effects on growth and progression.^8^ Cytotoxic T lymphocytes and natural killer cells inhibit the proliferation of thyroid cancer cells.^9^, ^10^ In contrast, tumor‐associated macrophages affect the progression and invasion of thyroid cancer.^11^, ^12^, ^13^ Considering the recent trials of immune checkpoint inhibitors for radioiodine‐refractory thyroid cancer, it is important to stratify patients according to the tumor immune context to determine treatment strategies, particularly compared to current options including tyrosine kinase inhibitors.^14^ In other words, it would be important to clinically identify immune‐rich type advanced thyroid cancer.

This study aims to identify the association between the glucose metabolism of thyroid cancer that can be assessed by FDG PET and the immune cells of the thyroid cancer microenvironment. Integrative analysis of FDG PET and RNA sequencing data was performed to understand the relationship between FDG uptake and the molecular characteristics of the TME of thyroid cancer. According to the findings, we hypothesized that intense FDG uptake in the tumor could be mediated by immune cells in the TME. To further elucidate the partitioning of glucose metabolism of cell types in the TME, publicly available single‐cell RNA sequencing (scRNA‐seq) data were used.

## 2 MATERIALS AND METHODS

### 2.1 Subjects

Twenty‐two patients with thyroid cancer who underwent FDG PET before surgery were previously enrolled for obtaining RNA‐seq from tumor tissues.^15^, ^16^ Since preoperative FDG PET for thyroid cancer are not routinely performed in Seoul National University Hospital, limited number of patients who underwent FDG PET for medical screening were included. The diagnosis of thyroid cancer was confirmed by histological analysis of surgery tissue. RNA sequencing data of thyroid cancer were obtained from the data repositories (European Genome Phenome Archive with accession number EGAS00001003540 and European Nucleotide Archive database with accession number PRJEB11591). All procedures were approved by the institutional review board of Seoul National University Hospital (H‐1903‐073‐1017, H‐1307‐034‐501) following the 1964 Helsinki Declaration. Informed consent was obtained from all individual participants included in the study. The clinical information of thyroid cancer patients is shown in Table 1.

**TABLE 1:** Clinicopathological characteristics of the 22 thyroid patients included in the study.

Variables		No. of available data
Gender (Male: Female)	7: 15	22
Age (years)	64.68 ± 13.92 (40–91)	22
Histology		
PTC/FVPTC/FTC/PDTC.ATC	4/3/7/8	22
Lymph node metastasis		
Yes/No/Unknown	7/6/9	22
Distant metastasis		
Yes/No	11/11	22
Stage		
1/2/3/4	5/3/0/14	22
SUV_max_	12.78 ± 12.48 (0.5–47.1)	22

Abbreviations: FTC, follicular thyroid cancer; FVPTC, follicular variant papillary thyroid cancer; PDTC.ATC, poorly differentiated thyroid cancer & anaplastic thyroid cancer; PTC, papillary thyroid cancer; SUV_max_, maximum standard uptake value.

### 2.2 Image acquisition and analysis

FDG PET scans were performed using dedicated PET/CT scanners of our center: Biograph mCT40 and mCT64 (Siemens Medical Solutions, Erlangen, Germany). As a routine clinical protocol, after 8 hours of fasting, the patients were intravenously injected with 5.18 MBq/kg (0.14 mCi/kg) of F‐18 FDG. The images from the thighs to the cranial vertex were obtained 60 min after the injection. PET data were reconstructed by an iterative algorithm. All PET images were interpreted using the dedicated software syngo.via (Siemens Medical Solutions, Erlangen, Germany). Imaging parameters, including the maximal standardized uptake value (SUV_max_), were evaluated by manually drawing a volume of interest over the thyroid lesion. To conduct the correlation analysis between transcriptome‐level features and imaging features, we utilized SUV_max_ as it is a widely accepted measure for tumor metabolism in clinical practice and represents the most metabolically active part of the tumor.

### 2.3 RNA sequencing data

Bulk RNA‐seq data of thyroid cancer tissues were processed according to the previous studies.^17^, ^18^ Briefly, read alignment and gene expression quantification were performed using STAR and HTSeq, respectively. The counts were normalized and log transformed by edgeR, to calculate log(cpm).^19^, ^20^ In addition, scRNA‐seq data of papillary thyroid cancer (PTC) were obtained from NCBI's Gene Expression Omnibus (GEO) Series accession number GSE184362 offered by a paper.^21^ We directly used gene counts uploaded by the authors. The gene expression data of 158,577 cells from 11 patients were downloaded. The data preprocessing was performed with Seurat (ver 4.1.0).^22^ The counts were log‐normalized with a scale factor of 10,000. Then, 2000 highly variable genes were identified using variance‐stabilizing transformation. The data were scaled based on the total read counts and mitochondrial read counts. Dimension reduction was performed by principal component analysis, and 30 dimensions were used for clustering. Cells were clustered using the *FindClusters* function with a resolution of 0.5. The labels of cell types were based on the marker genes in the literature. The scRNA‐seq data were embedded using uniform manifold approximation and projection (UMAP).

### 2.4 Gene signature scores

The glucose transporter (GLUT) feature was defined as the sum of *GLUT1* and *GLUT3*, as FDG uptake depends on two GLUT subtypes.^23^ We summed cpm values of the two gene expression data and log transformed. The glycolysis feature was defined by the Reactome pathway and Mootha pathway.^24^, ^25^ The tumor differentiation score (TDS) was also calculated with a set of specific genes of *DIO1*, *DIO2*, *DUOX1*, *DUOX2*, *FOXE1*, *GLUIS3*, *NKX201*, *PAX8*, *SLC26A4*, *SLC5A5*, *SLC5A8*, *TG*, *THRA*, *THRB*, *TPO*, and *TSHR*.^26^ The cytolytic feature was defined as the sum of *GZMA* and *PRF1*.^27^ The PD‐L1 expression was also evaluated by the gene expression of *CD274*. For bulk RNA sequencing data, the score of each signature was estimated by the sum of log‐expression values of genes. For scRNA‐seq data, the feature scores were estimated by the *AddModuleScore* function of Seurat using normalized gene counts.

### 2.5 Immune cell enrichment analysis

To evaluate the immune landscape of bulk RNA sequencing data, cell type enrichment scores were calculated. We used the tool EPIC (Estimating the Proportions of Immune and Cancer cells), which estimates the cell type enrichment score from predefined cell reference expression profiles of human tumor bulk RNA‐seq data.^28^ We assessed the enrichment scores of 8 cell types using the *EPIC* function in the EPIC package in R.

### 2.6 Statistical analysis

All data were analyzed by R (ver 4.1.1). Pearson's correlation test was used to evaluate the associations between two variables: SUV_max_ and signatures including glucose metabolism, TDS, cytolytic score, and the proportion of macrophages calculated by EPIC; and TDS of thyroid cells and signatures of glucose metabolism in myeloid cells.

## 3 RESULTS

### 3.1 FDG uptake correlated with metabolism signatures and immune cell enrichment in the TME

**FIGURE 1:**
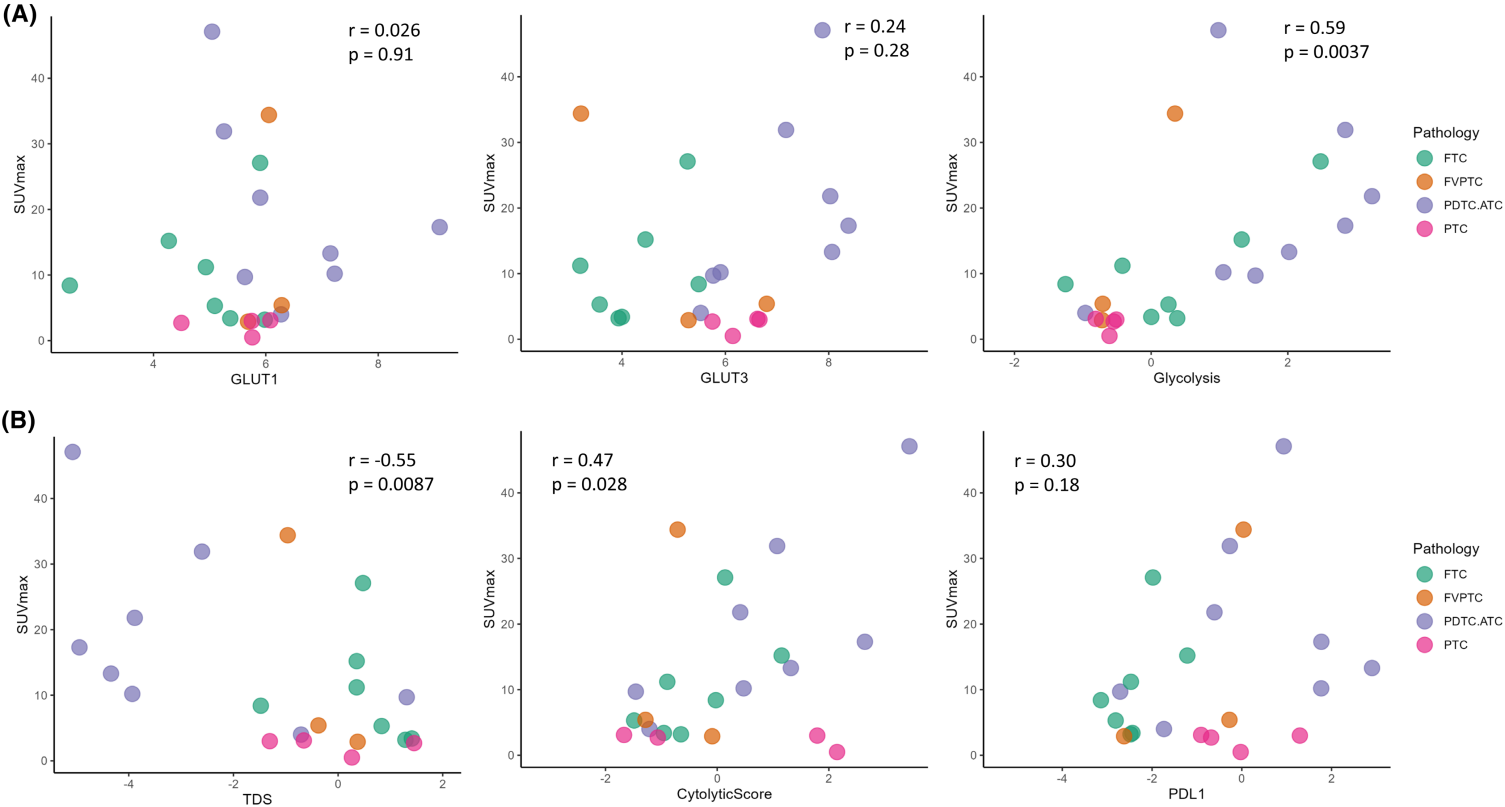
Scatter plots of SUV_max_ versus signatures of glucose metabolism, TDS, and cytolytic score. (A) Scatter plots of SUV_max_ versus glucose metabolism signatures including *GLUT1*, *GLUT3*, and glycolysis scores. All signatures of glucose metabolism showed positive correlations with SUV_max_ (*r* = 0.026, *p* = 0.91 for *GLUT1*; *r* = 0.24, *p* = 0.28 for *GLUT3*; *r* = 0.59, *p* = 0.0037 for glycolysis). (B) Scatter plots of SUV_max_ versus TDS and cytolytic score. TDS showed a negative correlation with SUV_max_ (*r* = −0.55, *p* = 0.0087), whereas cytolytic score was positively correlated with SUV_max_ (*r* = 0.47, *p* = 0.28). No significant correlation was found between SUV_max_ and PDL1 (*r* = 0.30, *p* = 0.18). SUV_max_: maximum standard uptake value; TDS: tumor differentiation score.

To begin with, we utilized gene expression data obtained from bulk RNA sequencing of thyroid cancer patients to estimate glucose metabolism scores, TDS, and cytolytic scores. Afterward, we compared SUV_max_ with these glucose metabolic profiles, TDS, and cytolytic scores to investigate the relationship between tumor metabolism and the TME. Although previous studies showed that expression of GLUTs was mainly positively correlated with FDG uptake in various tumor types,^29^ human thyroid cancer did not show significant correlations (*r* = 0.026, *p* = 0.91 for *GLUT1*; *r* = 0.24, *p* = 0.28 for *GLUT3*). On the other hand, the glycolysis score showed a positive correlation with SUV_max_ (*r* = 0.59, *p* = 0.0037 for glycolysis; Figure 1A). TDS, calculated using 16 genes related to thyroid tumor differentiation, was negatively correlated with SUV_max_ (*r* = −0.55, *p* = 0.0087), as expected by the ‘flip‐flop phenomenon’. As a representative value of the T and NK cell activity scores, the cytolytic score was significantly positively correlated with SUV_max_ (*r* = 0.47, *p* = 0.028). As another immune‐related marker, *PD*‐*L1* expression showed a weak positive correlation with SUV_max_, although the correlation did not reach statistical significance (*r* = 0.30, *p* = 0.18; Figure 1B).

**FIGURE 2:**
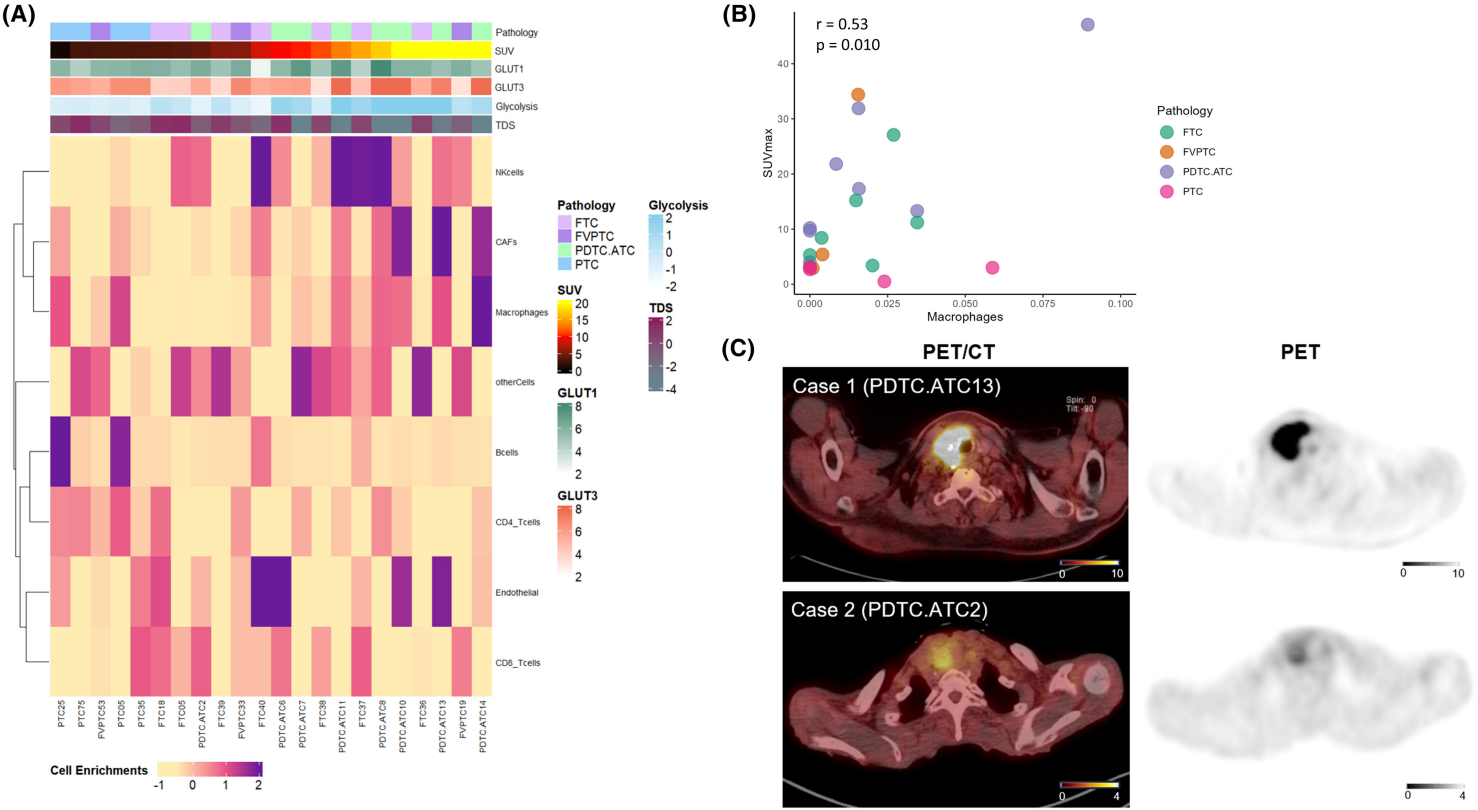
Enrichment scores of the 8 EPIC immune cell types. (A) Heatmap of EPIC enrichment scores of 8 immune cell types in 22 thyroid cancer tissues with F‐18 FDG PET/CT. A high SUV_max_ corresponded with high immune cell enrichment scores of macrophages and CAFs. (B) Scatter plots of SUV_max_ versus enrichment score of macrophages. Pearson's correlation analysis showed that a positive correlation was found between SUV_max_ and macrophage score (*r* = 0.53, *p* = 0.010). (C) Two representative F‐18 FDG PET/CT images with different FDG uptake. Case 1 is a 71‐year‐old woman with PDTC.ATC and Case 2 is a 91‐year‐old woman with PDTC.ATC. The SUV_max_ of the tumor was measured as 31.9 and 4.0, respectively. PTC: papillary thyroid cancer; FVPTC: follicular variant papillary thyroid cancer; FTC: follicular thyroid cancer; PDTC.ATC: poorly differentiated thyroid cancer & anaplastic thyroid cancer; TDS: tumor differentiation score; SUV_max_: maximum standard uptake value; CAF: cancer‐associated fibroblast.

A cell enrichment analysis was conducted to evaluate the immune cell population in the TME of thyroid cancer tissues (Figure 2A). Thyroid cancers with higher SUV_max_ exhibited higher scores for immune cell types including macrophages and cancer‐associated fibroblasts (CAFs). Another cell enrichment analysis, Xcell, showed that thyroid cancers with higher SUV_max_ had higher scores for phagocytes (Figure S1). A positive correlation was found between the enrichment level of macrophages and SUV_max_ (*r* = 0.53, *p* = 0.010; Figure 2B). Advanced thyroid cancers showed various FDG uptakes, as shown in representative cases (Figure 2C).

### 3.2 Single‐cell data revealed relatively high glucose metabolism signatures in immune cells in the TME

**FIGURE 3:**
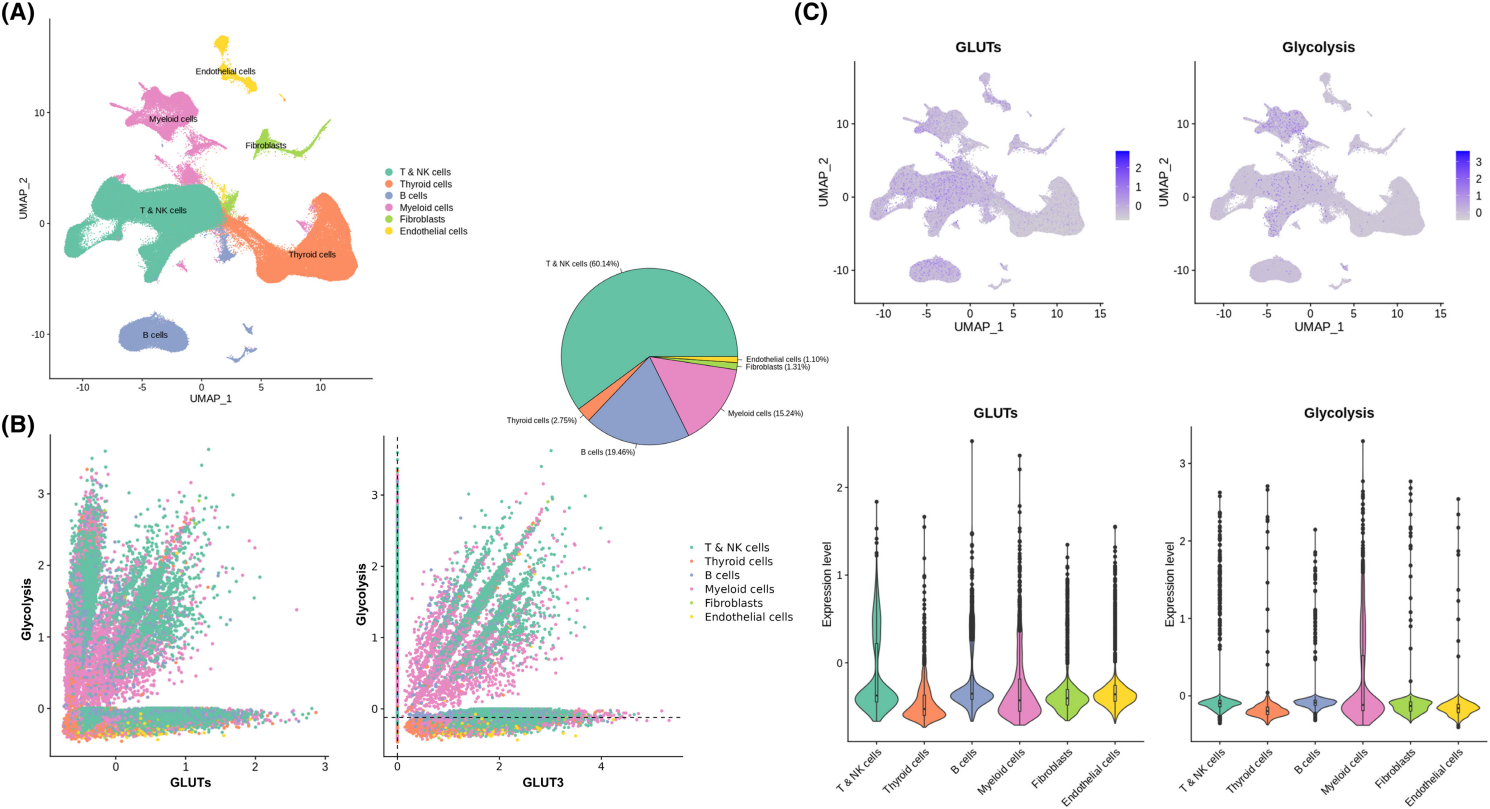
Glucose metabolic features from public scRNA‐seq of PTC. (A) A total of 158,577 cells from scRNA‐seq data clustered into 27 clusters and 6 cell types. (B) Scatter plot of glucose metabolic features. The dotted lines represent the median values of *GLUT3* and glycolysis scores. Among cells with *GLUT3* higher than the *GLUT3* median and glycolysis higher than the glycolysis median, the majority were T & NK cells, B cells, and myeloid cells (60.14% for T & NK cells; 19.46% for B cells; 15.24% for myeloid cells). (C) UMAP and violin plots with box plots representing GLUTs and glycolysis scores according to cell type. GLUTs and glycolysis scores were mainly expressed by T & NK cells, B cells, and myeloid cells. UMAP: uniform manifold approximation and projection.

Using publicly available scRNA‐seq data of human thyroid cancer, a total of 158,577 cells from 11 patients were clustered into 27 clusters and 6 cell types (Figure 3A). Cells were plotted according to the expression of GLUTs and glycolysis (Figure 3B). The proportion of cells with both high *GLUT3* and high glycolysis scores was evaluated. The major population was composed of T & NK cells, B cells, and myeloid cells, rather than thyrocyte‐origin cells, which mostly included thyroid cancer cells. The expression of GLUTs and glycolysis scores is also presented in UMAP and violin plots (Figure 3C). The distribution patterns of GLUTs and glycolysis scores were similar and were mainly upregulated in T & NK cells, B cells, and myeloid cells compared with thyrocyte‐origin cells.

**FIGURE 4:**
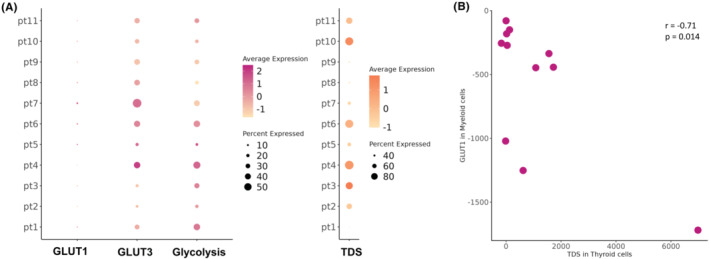
Glucose metabolic features of myeloid cells and TDS of thyroid cells. (A) Dot plot showing the average expression of glucose metabolic features in myeloid cells and TDS in thyroid cells from scRNA seq. (B) Scatter plot of *GLUT1* in myeloid cells versus TDS in thyroid cells. TDS in thyroid cells showed a negative correlation with *GLUT1* in myeloid cells (*r* = −0.71, *p* = 0.014). TDS: tumor differentiation score.

The average expression of glucose metabolic profiles of myeloid cells and TDS of thyrocyte‐origin cells were evaluated in each patient (Figure 4A). The expression of *GLUT1* was relatively low compared to *GLUT3* and the glycolysis score in myeloid cells of the TME. The previous EPIC analysis showed that myeloid cells, including macrophages, were enriched in thyroid cancers with a higher SUV_max_. Additionally, myeloid cells were the major cell types with high GLUTs and glycolysis, as shown in Figure 3. Therefore, we focused on the GLUTs of myeloid cells and examined their correlation with TDS in thyroid cells (Figure 4B). The TDS of thyrocyte‐origin cells was significantly negatively correlated with the *GLUT1* of myeloid cells (*r* = −0.71, *p* = 0.014). On the other hand, in thyrocyte‐origin cells, no significant relationship was found between the TDS and glucose metabolic signatures (Figure S2).

## 4 DISCUSSION

In this study, we investigated FDG PET and bulk RNA sequencing data of thyroid cancer patients to analyze the relationship between FDG uptake and TME features, including the immune cell enrichment score. In addition, the glucose metabolism of each cell type was dissected using scRNA‐seq data obtained from human thyroid cancer tissues. The results showed that relatively high glucose metabolism signatures were found in immune cells instead of thyrocyte‐origin cells, including cancer cells. This suggests that FDG uptake in thyroid cancer can be mainly mediated by immune cells of the TME. Furthermore, increased uptake associated with dedifferentiation could result from increased immune cells, particularly myeloid cells with high glucose consumption. Considering that both iodine scan and FDG PET are widely used to characterize thyroid cancer, the degree of FDG uptake can be used as a surrogate for immune metabolic functionality, particularly in advanced thyroid cancer.

From the point of view of a complex TME, increased glucose uptake is not only a characteristic of cancer cells. Increased metabolism, represented by aerobic glycolysis, is one of the hallmarks of cancer cells.^30^ Because of this property, FDG has long been widely used for cancer imaging to characterize tumor metabolism. However, single‐cell‐level studies have recently interrogated glucose metabolism in tumors and have shown hypermetabolism in immune cells. More specifically, glucose uptake per cell was higher in immune cells, particularly macrophages, than in cancer cells.^4^ Furthermore, cancer cells express *GLUT1*, while immune cells in the TME mainly express *GLUT3*,^31^ which shows higher glucose affinity than *GLUT1*.^32^ Therefore, FDG uptake of the gross tumor can be partly mediated by immune cells of the TME as well as cancer cells. As cancers with a high mutational burden and proliferative indices are related to increased glucose metabolism,^33^, ^34^ thyroid cancer, which shows a relatively low mutational burden and slow progression, would be expected to show relatively low glucose metabolism.^35^ However, high FDG uptake is usually identified on FDG PET, even in differentiated thyroid cancer.^36^, ^37^ This phenomenon can be explained by hypermetabolism of activated immune cells rather than increased metabolism due to aggressiveness of cancer cells in relatively slowly growing thyroid cancer. Our results support this idea. We found that FDG uptake in thyroid cancer was positively correlated with signatures of glycolysis and cytolytic scores. Aerobic glycolysis of tumors results from a higher demand for energy consumption,^38^ thereby presenting as FDG‐avid lesions. The cytolytic score was associated with cytotoxic T lymphocyte markers and regarded as a predictive marker for the responsiveness of immune checkpoint inhibitors.^27^ The positive correlation between cytolytic score and SUV_max_ implicates the immune activation status associated with FDG uptake. The negative correlation between TDS and SUV_max_ was compatible with previous studies since thyroid cancers with poor differentiation would need more glucose.^39^, ^40^

Moreover, immune cell enrichment analysis of bulk RNA sequencing showed that FDG‐avid thyroid cancers were enriched with macrophages (Figure 2). Xcell‐based cell enrichment analysis consistently showed that innate immune cells including phagocytes were enriched in FDG‐avid thyroid cancers (Figure S1). Myeloid cells have been shown to have the greatest capacity to take up and consume glucose within the TME.^4^ To identify the cell‐specific patterns related to FDG uptake in the thyroid cancer microenvironment, public scRNA‐seq data of PTC was adopted. Cells that showed high levels of both GLUTs and glycolysis were T & NK cells, B cells, and myeloid cells (Figure 3). Our bulk RNA sequencing data revealed that high FDG uptake in thyroid cancer was related to high GLUTs and low TDS. The TDS of thyroid cells did not correlate with the glucose metabolic profile of thyroid cells (Figure S2) but rather correlated with the GLUTs of myeloid cells (Figure 4). Thyroid cells with low TDS accompanied myeloid cells with high GLUTs, thus showing increased FDG uptake.

**FIGURE 5:**
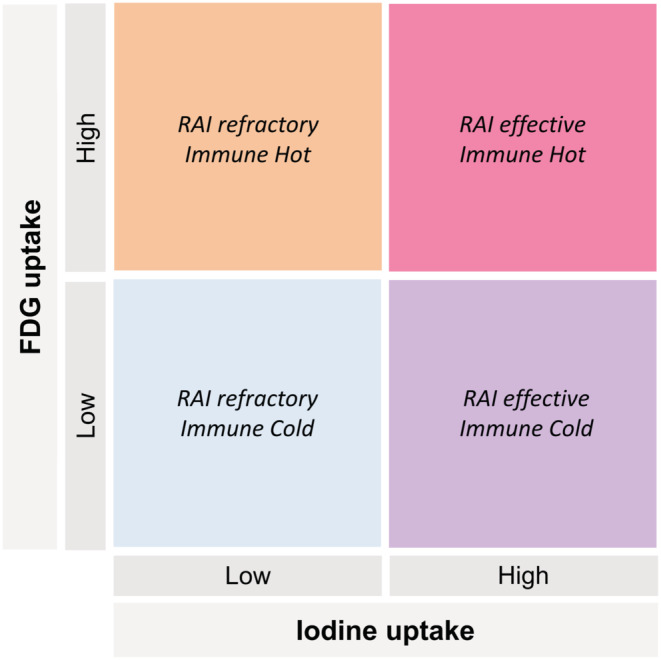
The diagram illustrates thyroid cancer types according to FDG and iodine uptake. Thyroid cancers can be divided into RAI refractory and RAI effective cancers depending on the degree of iodine uptake. In addition, thyroid cancers are further classified as immune cold and immune hot because FDG uptake represents the immune abundancy of the thyroid cancer microenvironment. RAI: radioactive iodine.

Our results of FDG uptake mediated by immune cells in thyroid cancer imply that stratification of thyroid cancer is possible using clinically available imaging studies. Of note, recent trials of immune‐oncology drugs for thyroid cancer^41^ characterizing the TME are critical because of the complexity of tumor immune properties. In this regard, immune cell enrichment and metabolic functionality reflected by FDG could be another axis of biological characteristics of thyroid cancer in addition to iodine avidity. Overall, we suggest that thyroid cancers could be further divided into immune hot or immune cold according to FDG‐avidity on F‐18 FDG PET, and hypothesize that thyroid cancers that do not follow the flip‐flop phenomenon may exist (Figure 5), which potentially applies to treatment stratification considering the option of immune‐oncology.

Although we analyzed the metabolic features of the thyroid cancer microenvironment with bulk and scRNA‐seq data, there are some limitations. First, the scRNA‐seq data were composed of only PTC patients. Although we performed thorough analyses on the metabolic characteristics of the thyroid cancer microenvironment, further studies encompassing scRNA‐seq data of other types of thyroid cancer are needed. In addition, we estimated the FDG uptake of 11 PTC patients in scRNA‐seq data based on transcripts of GLUTs and glycolysis scores, since 11 PTC patients did not have FDG PET/CT results. Direct matching FDG PET/CT results with scRNA‐seq data would provide supports for a correlation between glucose uptake and transcriptomic change in PTC. Moreover, expanding the transcriptomic analysis from the coding RNA to other components such as non‐coding RNA would help to comprehensively understand the metabolic profile of thyroid cancer since long non‐coding RNA has been known to influence the clinicopathologic features in PTC.^42^ Another limitation was that the amount of F‐18 FDG PET/CT data was small. Patients were not evenly enrolled in our study. Additional F‐18 FDG PET/CT of patients with bulk RNA sequencing data could clarify our results of the contribution of immune cells rather than thyroid cancer cells to FDG uptake. Finally, enriched immune cells in thyroid cancers could be resulted from undetected coexisting autoimmune thyroid disease,^43^, ^44^ despite our thorough inclusion of thyroid cancer patients without other thyroid disease. Therefore, further specific characterization of the immune cell infiltration in the thyroid cancer microenvironment is needed.

## 5 CONCLUSION

Integrative analyses of thyroid cancer have shifted attraction from cancer cells themselves to their TME, as understanding nutritional distribution in the cells of TME is fundamental to comprehending the metabolism of thyroid cancer. The cells that take up FDG in thyroid cancer are immune cells in the TME, rather than thyroid cells. FDG uptake was positively correlated with GLUTs and glycolysis, while it was negatively associated with differentiation. The scRNA‐seq data revealed that immune cells expressed high glucose metabolic features. Notably, GLUTs of myeloid cells showed a negative association with tumor differentiation. Therefore, thyroid cancer can be divided into immune cold or immune hot subtypes according to FDG uptake. We expect that the immune‐based classification of thyroid cancers could be applied to stratify patients using FDG PET/CT and provide appropriate treatment.

## AUTHOR CONTRIBUTIONS

**Hoon Young Suh:** Formal analysis (lead); investigation (lead); writing – original draft (equal). **Hongyoon Choi:** Conceptualization (lead); data curation (lead); software (lead); supervision (lead); writing – original draft (equal). **Sun Wook Cho:** Validation (supporting). **Jin Chul Paeng:** Writing – review and editing (supporting). **Gi Jeong Cheon:** Funding acquisition (equal); writing – review and editing (supporting). **Young Joo Park:** Resources (supporting); validation (supporting). **Keon Wook Kang:** Writing – review and editing (supporting).

## CONFLICT OF INTEREST STATEMENT

Hongyoon Choi is a co‐founder and CTO of Portrai, Inc. Sun Wook Cho is a co‐founder and CEO of cellus, Inc. The authors have declared that no competing interest exists.

## ETHICS STATEMENT

All procedures performed in studies involving human participants were in accordance with the ethical standards of the institutional and/or national research committee and with the 1964 Helsinki declaration and its later amendments or comparable ethical standards.

## CONSENT TO PARTICIPATE

Informed consent was obtained from all individual participants included in the study.

## Supporting information


Figure S1.

Figure S2.

## Data Availability

The RNA sequencing data of this study are available in the European Genome Phenome Archive at https://www.ebi.ac.uk/ega/ with accession number EGAS00001003540, reference number 16 and the European Nucleotide Archive database at https://www.ebi.ac.uk/ena/browser/ with accession number PRJEB11591, reference number 15. The scRNA‐seq data can be openly available in GEO database at https://www.ncbi.nlm.nih.gov/geo/ with accession number GSE184362, reference number 21.
