# Supplementary material for: FDG uptake reflects an immune‐enriched subtype of thyroid cancer: Clinical implications of imaging‐based molecular characterization
Source: Cancer Med. 2023 Jul 19;12(16):17068–77. doi: 10.1002/cam4.6350 (PMC10501276; doi:10.1002/cam4.6350)
Supplement: Supplementary file 1 — Figure S1. [file CAM4-12-17068-s001.pdf]

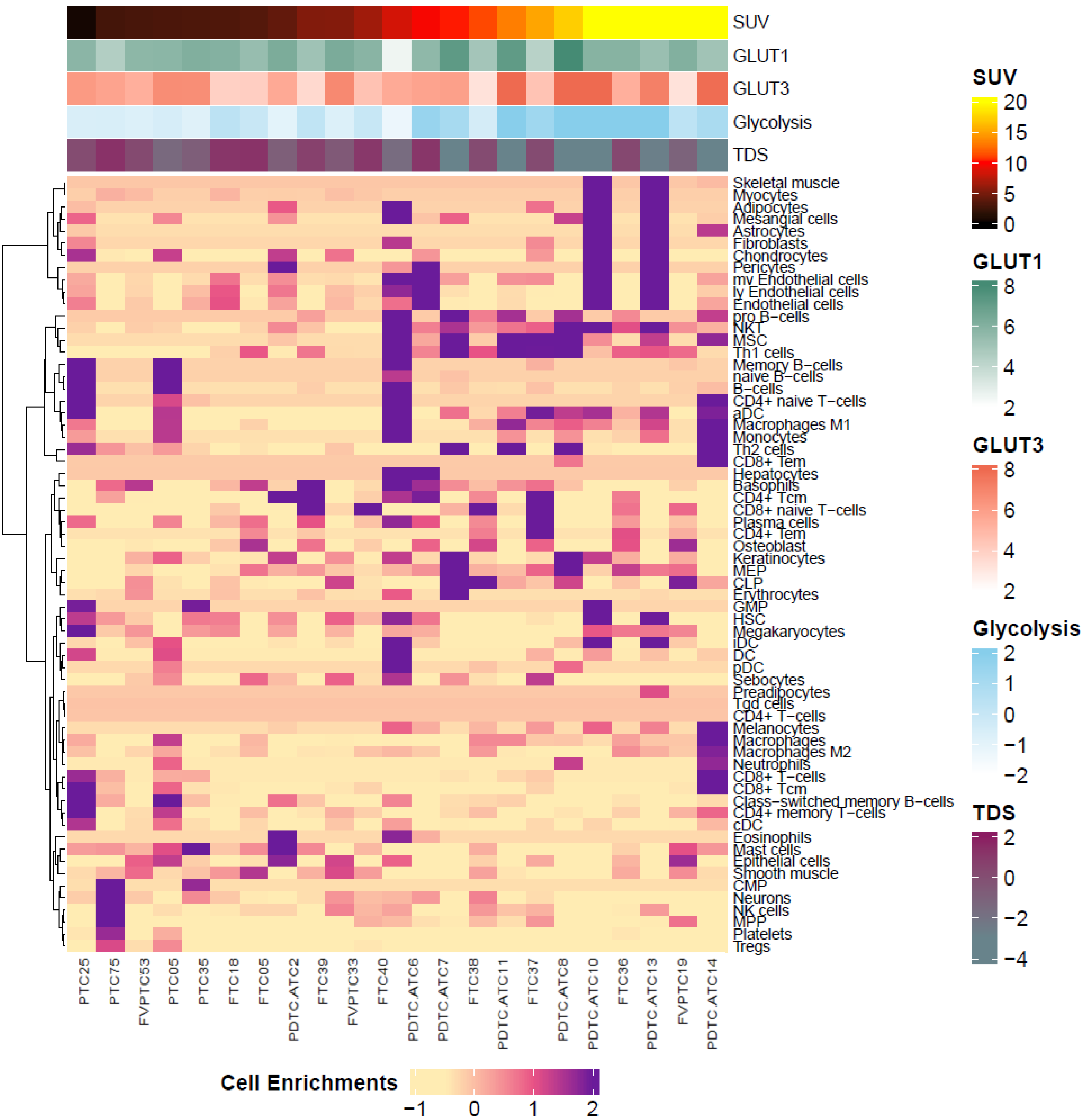

Supplementary Fig. 1 Enrichment scores of the Xcell immune cell types Heatmap of Xcell enrichment scores of 64 immune cell types in 22 thyroid cancer tissues with F-18 FDG PET/CT. A high SUVmax corresponded with high immune cell enrichment scores of phagocytes. PTC: papillary thyroid cancer; FVPTC: follicular variant papillary thyroid cancer; FTC: follicular thyroid cancer; PDTC.ATC: poorly differentiated thyroid cancer & anaplastic thyroid cancer; TDS: tumor differentiation score.
