# Supplementary material for: FDG uptake reflects an immune‐enriched subtype of thyroid cancer: Clinical implications of imaging‐based molecular characterization
Source: Cancer Med. 2023 Jul 19;12(16):17068–77. doi: 10.1002/cam4.6350 (PMC10501276; doi:10.1002/cam4.6350)
Supplement: Supplementary file 2 — Figure S2. [file CAM4-12-17068-s002.pdf]

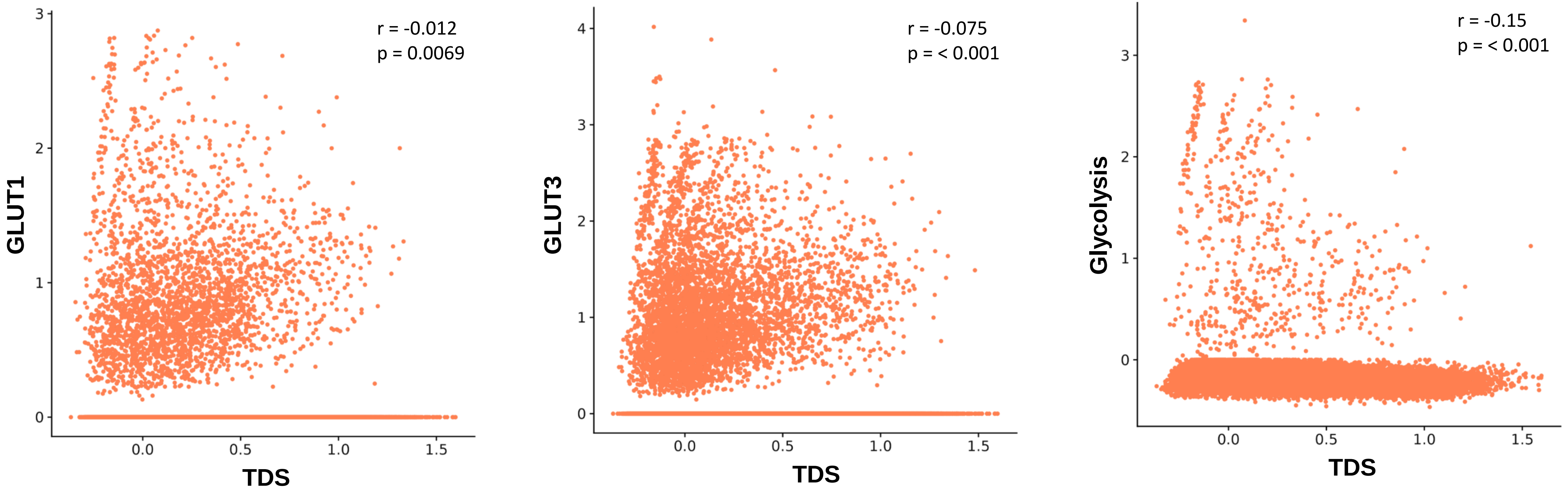

Supplementary Fig. 2 Scatter plots of TDS versus signatures of glucose metabolism in thyrocyte-origin cells Scatter plots of TDS versus glucose metabolism signatures including *GLUT1*, *GLUT3*, and glycolysis scores. All signatures of glucose metabolism showed no correlations with TDS (r = -0.012, p = 0.0069 for *GLUT1*; r = -0.075, p = <0.001 for *GLUT3*; r = -0.15, p = <0.001 for glycolysis). TDS: tumor differentiation score.
